# Chemogenetic interactions in human cancer cells

**DOI:** 10.1016/j.csbj.2019.09.006

**Published:** 2019-10-26

**Authors:** Medina Colic, Traver Hart

**Affiliations:** a Department of Bioinformatics and Computational Biology and Department of Cancer Biology, The University of Texas MD Anderson Cancer Center, Houston, TX, USA; b UTHealth Graduate School of Biomedical Sciences, The University of Texas MD Anderson Cancer Center, Houston, TX, USA

**Keywords:** CRISPR, Chemogenetic screens, Drug-gene interactions

## Abstract

Chemogenetic profiling enables the identification of genes that enhance or suppress the phenotypic effect of chemical compounds. Using this approach in cancer therapies could improve our ability to predict the response of specific tumor genotypes to chemotherapeutic agents, thus accelerating the development of personalized drug therapy. In the not so distant past, this strategy was only applied in model organisms because there was no feasible technology to thoroughly exploit desired genetic mutations and their impact on drug efficacy in human cells. Today, with the advent of CRISPR gene-editing technology and its application to pooled library screens in mammalian cells, chemogenetic screens are performed directly in human cell lines with high sensitivity and specificity. Chemogenetic profiling provides insights into drug mechanism-of-action, genetic vulnerabilities, and resistance mechanisms, all of which will help to accurately deliver the right drug to the right target in the right patient while minimizing side effects.

## 1 Introduction

A major goal of precision medicine is the development of selective anticancer drugs that effectively target tumors while minimizing the side effects associated with conventional chemotherapy. Development of anticancer drugs and repurposing existing drugs is often driven by identifying tumor-specific molecular alterations. To understand these alterations and exploit them as drug targets, it is necessary to study how they define a molecular context that allows sensitivity or resistance to particular compounds [1]. Integration of traditional genetic approaches with the new wealth of genomic information from both human and model organisms established techniques by which drugs can be profiled for their ability to selectively kill cells in a molecular context same as those in tumors. Such profiling allows for identifying genes whose mutations produce the desired therapeutic outcome, and using that knowledge it is possible to identify and validate new drug targets. In addition, this genetic approach for drug discovery also allows us to use mutation as a model of an ideal drug. Through gene knockouts, we can eliminate particular protein's functions, genetically modeling a perfect drug for that target. In this review we focus on findings specific to chemogenetic methods; a similar report [2] includes additional information about other experimental approaches, including affinity-based and comparative profiling methods.

### 1.1 Chemogenetics in yeast

A 1983 study done in *Saccharomyces cerevisiae* was one of the first chemogenetic experiments, characterizing the previously unknown metabolic target of a therapeutic agent. Using one yeast strain and existing yeast clone pool, Rine et al. isolated genes whose products are sensitive to specific inhibitors: compactin, tunicamycin and ethionine (targeting the unrelated biosynthesis pathways of sterol biosynthesis, glycoprotein biosynthesis, and AdoMet biosynthesis, respectively). The approach used in this study was based on the premise that increasing the copy number of a gene increases the amount of gene product [3]. In a landmark study describing genetic approaches for discovering cancer drugs by Hartwell et al. [4], a panel of *Saccharomyces cerevisiae* DNA damage response mutants were used to test for genotype-dependent variation in drug response. The increased sensitivity of specific yeast mutants to particular types of DNA damage – e.g. the hypersensitivity of DSB repair mutants to topoisomerase II poison mitoxantrone – supported the then-revolutionary notion that chemogenetic screening could match chemotherapeutic compounds to the tumor genotypes where they would be most effective.

Induced haploinsufficiency is another approach used for genomic profiling of drug sensitivities. *Saccharomyces cerevisiae* was again the model organism of choice due to the experimental tractability of the yeast genome, which, coupled with a sequenced yeast genome, allowed for the systematic construction of heterozygous deletion strains in any essential or nonessential gene [5], [6], [7]. In this study Giaever et al. tested six heterozygous strains carrying deletions in known drug targets (*HIS3*, *ALG7*, *RNR2*, *TUB1*, *TUB2*, *ERG11*) for induced haploinsufficiency. All of the tested strains showed induced haploinsufficiency in the presence of at least one drug, defining a class of genes that exhibit induced context-dependent haploinsufficiency. In the same study, drug-sensitivity profiling of 233 heterozygous strains in the presence of tunicamycin revealed three drug-sensitive loci: *ALG7* (member of a protein glycosylation and a known target of tunicamycin), *YMR266W* encoding a protein with homology to the multi-facilitator superfamily and *YMR007W* encoding a protein with unknown function [8]. Within a few years, the same group developed a nearly complete collection of gene-deletion mutants (96% of annotated open reading frames, ∼6000 genes) in *S. cerevisiae* [9]. In this study the strains were constructed with molecular bar codes to permit the identification and extraction of individual mutant sensitivities from genome-wide competitive growth in a single culture. In 2004, three other groups investigated the sensitivity of large-scale yeast strains to small molecule inhibitors or drugs [10], [11], [12]. Tucker and Fields investigated the sensitivity of 4800 haploid yeast strains to ibuprofen, using a genetic-array-based method [10]. Using the same genetic arraying approach Baetz et al. screened 5000 heterozygous yeast mutants for sensitivity to dihydromotuporamine C, a compound used in preclinical development as an inhibitor of metastasis at the time [11]. Lum et al. profiled 78 different drugs, the majority of which are approved by the FDA and are considered to have well-characterized targets, in 3500 heterozygous yeast diploid strains using the barcoding method [12]. Studies with such high-throughput chemical screening genetic-array-based or barcoding methods presented approaches to identify gene-drug and pathway-drug interactions on a previously unavailable scale. Hillenmeyer et al. performed 1144 chemical genomic assays on the yeast whole-genome heterozygous and homozygous deletion pool to reveal the phenotypes for the nonessential portion of the yeast genome (∼80%). They have found that 97% of gene deletions exhibited growth phenotype, suggesting that almost all genes are essential for optimal growth in at least one condition [13]. In parallel, Hoon et al. integrated three genome-wide gene dosage assays (homozygous deletion mutants, heterozygous deletion mutants, and genomic library transformants) to measure the effect of small molecule in yeast. Their study confirmed that this integrated approach improves the sensitivity and specificity of small-molecule target identification, and allows the identification of both potential targets and structure-activity relationships. A more detailed review of yeast chemogenetics can be found in similar studies [14], [15], [16], [17].

Despite the advantages of yeast as a model organism for chemogenetic methods, it has limitations in identifying the molecular targets of drug candidates, and drug-gene interactions for use in human cells. Many genes are not conserved between yeast and humans, and conserved genes have frequently expanded into large paralog families in mammals. In addition, as unicellular organisms, yeast can’t recapitulate the complex cellular organization of a human and are often a poor model for tissue-specific drug response in higher eukaryotes. Thus, utilizing the findings and technology from these yeast studies, efforts continued towards developing adequate approaches in human cells.

### 1.2 RNAi, the first step toward chemogenetics in human cells

Within a few years of these seminal yeast studies, researchers were using RNA interference (RNAi) in arrayed small interfering RNA (siRNA) and pooled library short hairpin RNA (shRNA) to perturb gene expression in mammalian cell lines on a large scale. After its discovery in *Caenorhabditis elegans*, RNAi showed great potential for use in guiding development of therapeutic agents in human cells [18], [19], [20]. Identifying novel targets of a compound, novel pathways that affect the activity of a compound and potential biomarkers became available via screening small molecules (drugs, chemical compounds) against siRNA libraries [21]. Screening kinase and phosphatase libraries of siRNAs discovered genes involved in resistance to standard-of-care chemotherapies [22], [23], [24]. Large-scale shRNA libraries were also used for chemogenetic screening to map out genetic modifiers that enhance or suppress the activity of small molecules. With widespread use, however, the method’s major technical shortcomings were revealed: incomplete target knockdown, poorly predicted off-target effects, and an overall failure to appreciate or effectively model the experimental noise left researchers – and drug developers – highly skeptical of RNAi-generated leads [19], [25], [26], [27], [28], [29].

One approach to overcome the low signal-to-noise problem posed by early RNAi applications was to vastly expand the number of reagents applied per gene. Whereas a typical pooled library screen had used ∼5 shRNA hairpins targeting each gene [22], [30], [31], Bassik et al. [32] developed an “ultracomplex” library with 25 shRNA hairpins targeting each of nearly 19,000 genes. This massive library, coupled with high coverage – each hairpin represented, on average, by over 1000 cells in the screen (“1000× coverage”) – was used to probe the cellular response to ricin toxin in K562 chronic myelogenous leukemia (CML) cells. Pulsed treatment with ricin at LD_50_ over several days revealed dozens of genes in well-described pathways whose knockdown led to resistance or sensitization to the poison. This ultracomplex shRNA screening platform was subsequently used to identify the specific target of a previously uncharacterized molecule. In November 2013, Matheny et al reported that nicotinamide phosphoribosyl transferase (NAMPT), a key enzyme in NAD biosynthesis, had been identified as the primary target of compound STF-118804 by screening MV411 acute monocytic leukemia (AML) cells with high doses of the compound and looking for synthetic lethal drug-gene interactions [33].

Although the efficacy of these examples is impressive, the scale of the experiments is daunting. The ultracomplex library is comprised of 9 subpools of 55,000 shRNA hairpins each. To achieve 1000× coverage requires the successful transduction of nearly 500 million cells; at a starting multiplicity of infection (MOI) of 0.3, typically used to ensure that most cells are infected by a single shRNA-carrying virion, this implies a starting population 1.5 × $10^{9}$cells [20]. Given that cells are grown for at least two weeks after transduction and selection, it is clear why suspension cell lines, which can be propagated at high density, were preferred for these assays.

### 1.3 The CRISPR/Cas9 revolution and chemogenomic applications

**Fig. 1:**
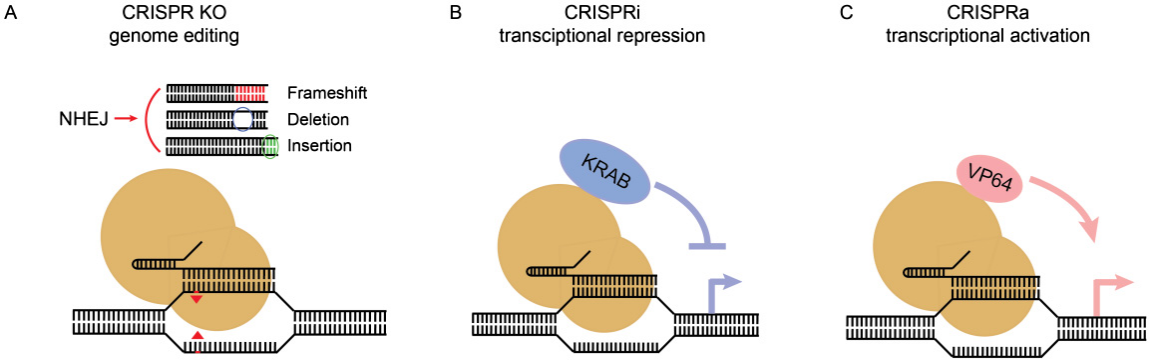
CRISPR technologies to perturb gene functions in mammalian cells for pooled genetic screens. CRISPR loss-of-function technologies include A) CRISPR knockout (KO) and B) CRISPR interference (CRISPRi). A) Cas9-mediated DNA cleavage is directed to the coding region of a gene by a single guide RNA (sgRNA) and it results in error-prone repair by nonhomologous end joining pathways (NHEJ), and as a consequence of that gene function is disrupted (when indels and especially frame shifts are introduced). B) Catalytically dead Cas9 (dCas9) is fused to a transcriptional repressor domain (e.g. KRAB) and as that is recruited to the transcription start site (TSS) of a gene specified by an sgRNA, to repress its transcription. CRISPR gain-off-function technology is C) CRISPR activation (CRISRPa). C) dCas9 is fused with transcriptional activation domain(s) (e.g. VP64) and recruited to a given gene’s TSS, to activate its transcription.

The development of the ultracomplex RNAi library was largely concurrent with the discovery of the CRISPR/Cas9 RNA-guided endonuclease system and its adaptation to mammalian genome engineering, reported in early 2013 [34], [35]. Cas9 introduces a double-strand break at a locus specified by a guide RNA, triggering DNA endogenous repair mechanisms that in protein coding regions frequently result in frameshift mutations that result in a loss of function of the encoded protein [36] (Fig. 1A).

**Fig. 2:**
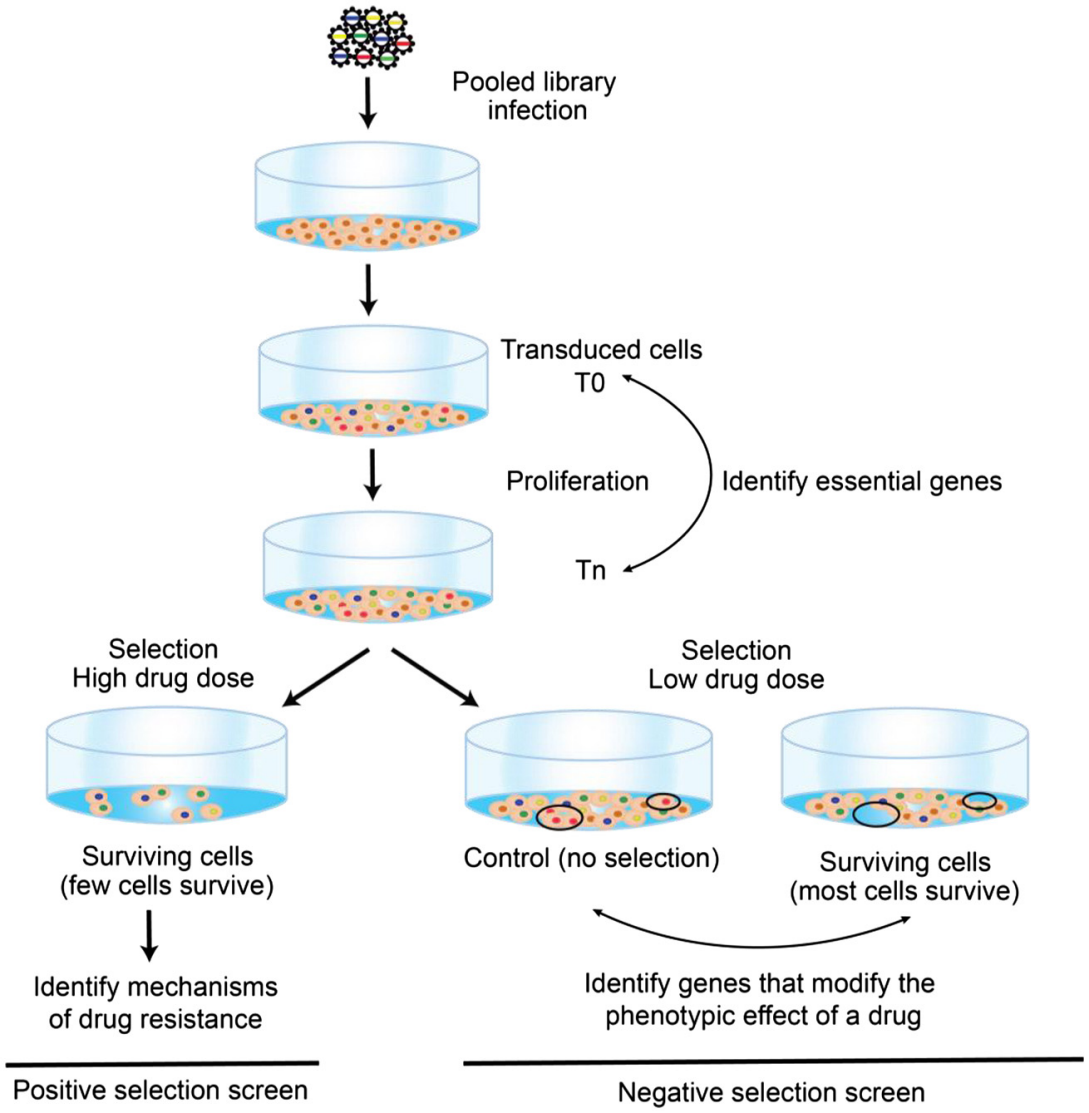
Experimental design for a whole genome CRISPR screen. In a pooled library CRISPR screen, cells are transduced with a pooled CRISPR library. Successfully transduced cells are sampled (T0) and grown for several doublings. At Tn cells are sampled again. Genomic DNA is extracted from T0 and Tn cells, PCR-amplified and sequenced using NGS. To identify essential genes (i.e. genes whose knock-out results in a fitness defect) abundance of each sgRNA at Tn is compared to abundance of each sgRNA at T0. Genome-wide CRISPR screens can be divided into two classes, positive and negative selection. In a positive screen, the goal is to identify those cells that survive post-selection (e.g. drug treatment). The selective pressure must be strong enough that most of the cells die, removing their sgRNAs from the population, and only a small fraction survives. After the surviving cells are collected, their plasmids are PCR-amplified and sequenced using NGS to identify their target gene. In a negative screen, the goal is to identify those cells that do not survive the selection mechanism. Two sets of cells are infected, one set is subject to selection (e.g. drug treatment) while the other set serves as a non-selected (i.e. non-treated) control. These two populations are then sequenced using NGS to determined which sgRNAs have been depleted by selection.

Within a year, two groups had reported using the CRISPR/Cas9 system for large-scale gene knockout studies in human cells (Fig. 2). Wang et al targeted 7000 genes with 10 guide RNA (gRNA) per gene in near-haploid KBM7 CML cells [37]. As a proof of concept, they screened for survival in the presence of 6-thioguanine (6TG), a compound that introduces DNA lesions that, in cells with a functional mismatch repair pathway, causes cell cycle arrest. All four genes involved in basis mismatch repair (*MLH1*, *MSH2*, *MSH6*, *PMS2*) were identified with high specificity. A follow-up screen in leukemic HL60 cells in the presence of etoposide, a topoisomerase II poison, identified *TOP2A* as well as cell cycle checkpoint kinase *CDK6* as resistance genes.

At the same time, Shalem et al reported their own pooled library CRISPR screen, with 65 k gRNA targeting 18,080 protein-coding genes. Exposing transduced BRAF^V600E^ A375 melanoma cells to the *BRAF* inhibitor vemurafenib yielded enrichment for known resistance gene *NF1*, as well as a handful of other genes including *NF2* [38]. Interestingly, in a subsequent whole-genome screen for fitness genes in this same cell line [39], NF2 knockout appears to confer a fitness advantage on A375 cells [40], consistent with its known role as a tumor suppressor. These two proof-of-concept studies solidified the CRISPR/Cas9 system as a sensitive drug-screening tool for mammalian cells, at least for positive selection screens (Fig. 2). Moreover, both examples required a significantly smaller footprint that the RNAi studies, with 70 k libraries screened at 300–500× coverage (i.e. ∼35 × 10^6^ cells grown and passaged).

A subsequent head-to-head evaluation of ultracomplex RNAi and small-scale CRISPR knockout by Deans et al, K562 cells were transduced with either the shRNA library or a CRISPR knockout library targeting the entire human protein-coding genome (∼4 sgRNAs per gene and ∼2000 negative control sgRNAs). In both screens, transduced cells were cultured in the presence or absence of GSK983, a compound with broad-spectrum antiviral activity [41]. This study determined that GSK983 inhibits dihydroorotate dehydrogenase *DHODH* to block virus replication and cell proliferation, and additionally purported to demonstrate the mechanistic difference between CRISPR and shRNA screens. A widely discussed conclusion from this study was shRNA might better mimic the effects of drug exposure, since shRNA induces only partial knockdown of its targets and CRISPR-mediated knockout results in complete loss-of-function alleles, and might therefore represent a better screening approach for therapeutic targets. However, the data for this conclusion are weak: the shRNA screen is performed at extremely large scale, while the CRISPR screen is much lower coverage using an early-generation library for which no overall screen quality metrics are published. Indeed, in light of subsequent data, it seems likely that the CRISPR screen showed a high false negative rate due to experimental and/or informatic idiosyncrasies rather than technological limitations.

Indeed just a few months later, a CRISPR-mediated chemogenetic interaction screen was reported that identified the specific target of an uncharacterized compound, LB-60-OF61, that selectively killed cancer cells [42]. A 90 k gRNA library was transduced into adherent HCT116 colorectal cancer cells at 1000× coverage, expanded, and split into three treatment arms. Control cells and cells treated with the compound at IC30 and IC50 were cultured for three weeks, with samples collected at three timepoints. This comprehensive experimental design allowed not only the identification of the target of the compound *(NAMPT*) but also the dynamics of hits over time and across experimental conditions. Of particular interest in this experiment is the clear demonstration of decreasing signal to noise as library coverage is reduced from 1000× to 1/2, 1/4, and 1/8 of that target value. The same experimental design also identified the endoplasmic reticulum-localized signal peptidase as the target of cavinafungin, potent and selectively active compound against the Zika virus [43].

Continued improvement in CRISPR reagent design [44], [45] and corresponding refinement of experimental methods has broadened the applicability of CRISPR-mediated chemogenetic interaction screening (Fig. 2). One recent study brings the arc of chemogenetics full circle: a screen for genetic modifiers of *PARP* inhibitors to identify the genetic backgrounds in which these new drugs might be therapeutically useful. Chemogenetic screens using the 90 k TKOv1 library [46] were carried out at ∼200× library coverage in immortalized human retinal epithelial RPE1-hTERT cells, HeLa cervical cancer cells, and SUM149PT cells originating from a triple-negative breast cancer and carrying a hemizygous *BRCA1* frameshift mutation. Treatment with olaparib identified a high-confidence set of 73 genes whose knockouts increase sensitivity to *PARP* inhibitors. In addition to an expected enrichment for genes related to homologous recombination and repair, this study also discovered that mutations in all three subunits of the ribonuclease H2 complex sensitized cells to *PARP* inhibition, and characterized the novel role of this protein complex in DNA repair. Further screens in three independent cell lines (293A, HCT116, and MCF10A) treated with AZD6738, a highly selective inhibitor of DNA damage checkpoint kinase ATR, identified genes whose loss makes tumor cells hypersensitive to *ATR* inhibition – and interestingly also demonstrated RNASEH2 synthetic lethality [47]. When results from these ATRi screens were compared with another set of ATRi screens (VE-821 as the *ATR* inhibitor in HCT116, HeLa, and RPE1 hTERT TP53^−/−^; AZD6738 as *ATR* inhibitor in RPE1 hTERT TP53^−/−^) a set of 11 genes were found as hits in at least 4 out of 7 screens indicating that they are likely to modulate the response to *ATR* inhibition independently of cellular context [48].

### 1.4 The role of CRISPRa and CRISPRi in chemogenetic screening

RNA-guided, CRISPR-mediated genome editing is not limited to gene knockouts induced by Cas9 double strand breaks. A nuclease-deficient Cas9 (dCas9) fused with a transcriptional activation or repression domain can be targeted to a gene promoter to activate (CRISPRa) (Fig. 1B) or inhibit (CRISPRi) gene transcription [49] (Fig. 1C). Gilbert et al. developed massive CRISPRi and CRISPRa libraries (206 k reagents targeting 20,898 transcriptional start sites of 15,977 genes) and deployed them at huge scale (3750× coverage – nearly 800 × 10^6^ cells) – again in suspension K562 cells – to identify fitness genes as well as toxin response. In the presence of a chimeric diphtheria/cholera toxin, both CRISPRi and CRISPRa screens were able to classify both sensitizers and suppressors of toxicity [49]. A smaller-scale screen (1000× coverage) in the same cells with candidate chemotherapeutic agent rigosertib identified a microtubule-destabilizing signature as its mechanism of action [50]. In parallel, Feng Zhang and colleagues reported the identification of genes whose overexpression in A375 melanoma cells gave rise to resistance against a BRAF inhibitor, an independent validation of the CRISPRa screening platform [51]. A study including all three approaches (CRISPRko, CRISPRi and CRISPRa) in the context of genome-wide screens to identify drivers of resistance and sensitivity to the BRAF inhibitor vemurafenib [52] yields results consistent with the previously published datasets [23], [28]. The CRISPRko and CRISPRi data sets are highly similar, leading the authors to suggest that a CRISPRi might be a more sensitive screening platform for some targets and that it might overcome some of the flaws of CRISPRko screens (e.g. sensitivity to copy-number amplification at the target locus). The CRISPRa data set from this study overlaps substantially with earlier mentioned CRISPRa data [51], and allows cross validation of hits identified from the CRISPRi screen. All three approaches have their unique strengths and disadvantages, however, this study emphasized that the precision of knock out screens is greatly enhanced when combining CRISPRi and CRISPRa screens in the same cells [52].

With CRISPR-based gene knockout and gene activation methods available, a few groups combined CRISPRko and CRISPRa screens to study gene functions, gene networks and uncover novel drug targets [53], [54], [55]. Prior developing and optimizing the orthogonal CRISPR system, Boettcher et al. identified genes whose activation can alter imatinib drug response. The study reports that main advantage of the gain-of-function approach (activation screen) used here, as opposed to more common loss-of-function approach, is that genes exhibiting no or very low expression can also be investigated. Indeed, out of 332 hits from this activation screen, 21% were not expressed in K562 cells in which the screen was performed, suggesting that imatinib-responsive genes could be identified from genes with a broad range of expression levels [55]. Najm et al. developed a lentiviral vector and cloning strategy to generate high-complexity pooled dual-knockout libraries (Big Papi – paired S. aureus and S. pyogenes Cas9 endonucleases for independent perturbations) to identify synthetic lethal and buffering gene pairs across multiple cell types (A375, Meljuso, HT29, A549, 786O, and OVCAR8). Identified hits include interactions between *MAPK* pathway genes, apoptosis genes, *AKT* paralogs and *BRCA* and *PARP* genes. To confirm the synthetic lethal interactions between anti-apoptotic genes, including synthetical lethality between *BCL2L1* and *MCL1*, and *BCL2L1* and *BCL2L2* (an interaction not observed prior to this study) authors used various small molecule inhibitors of these proteins. They confirmed the synthetic lethal interaction between *MCL1* and *BLC2L* by all combinations of drug-gene perturbation, and further demonstrated synergy of a two-drug cocktail [54]. A preprint from this year describes another study where similar idea of combinatorial screens was utilized to develop a ‘one-to-all’ approach which accommodates screening in isogenic mammalian cell lines without single cell cloning [56]. This approach is based on two vectors: the first vector, to which authors refer as “anchor” vector, delivers *S. pyogenes* Cas9, and a guide compatible with *S. aureus* Cas9; the second vector delivers *S. aureus* Cas9 and a guide cassette compatible with *S. pyogenes*, which is used to deliver the library of choice. A guide targeting gene of interest (“anchor gene” in their parlance, but frequently called “query gene” in the yeast genetic interaction nomenclature), is cloned into the anchor vector, and the population of cells expressing this vector is expanded. The authors argue that no editing will occur after this transduction because the single guide RNA of one bacterial species is paired with the Cas9 endonuclease of the other. In the next step, the library of choice is introduced, with the expectation that each cell will generate approximately simultaneous knockout of both the anchor gene and the gene targeted by the library. To test this approach, authors used widely studied and characterized genes: *BCL2L1*, *MCL1*, and *PARP1* as anchor genes, and cell lines: Meljuso, OVCAR8, A375 and Hap1. To validate detected genetic interactions with anchor genes, authors have performed chemogenetic screens using small molecules targeting these anchor genes (A-1331852 BCL2L1i, S63845 MCL1i, olaparib and talazoparib PARPi) (Table 1).

**Table 1:** Genome-Scale CRISPR-mediated Chemogenetic Screens in Human Cells.

Approach	Guides/gene	Genes	Cell Type	Phenotype [Reference]
CRISPRko	(Sabatini) 10	7114	Near-haploid KBM7 and pseudo-diploid HL60 leukemia cells	Resistance to thioguanine and etoposide [37]
CRISPko	(GeCKO) 3-4	18,080	A375 melanoma cells	Resistance to vemurafenib [38]
CRISPRko	(TKOv1) 6	17,661	RPE1-hTERT, HeLa and SUM149PT cells	Sensitivity and resistance to olaparib [46]
CRISPRko	(TKOv3) 4	18,053	293A, HCT116 and MCF10A cells	Sensitivity and resistance to ATR inhibition [47]
CRISPRko	(TKOv1) 6/(TKOv3) 4	17,661	HeLa, HCT116, RPE1 hTERT TP53−/− cell	Resistance to ATR inhibition [48]
CRISPRko	(custom) 5	18,080	HCT116 cells	Sensitivity and resistance to NAMPT inhibitor [42]
CRISPRko	(custom) 5	18,080	HCT116 cells	Sensitivity and resistance to cavinafungin [43]
CRIPSRi	10	49	K562 leukemia cells	Sensitivity to AB toxin ricin [43]
CRIPSRi	10	15,977	K562 leukemia cells	sensitivity to rigosertib [50]
CRIPSRa	10	49	K562 leukemia cells	sensitivity to AB toxin ricin [49]
CRISPRa	10	15977	K562 leukemia cells	Sensitivity to rigosertib [50]
CRISPRa	3	23,430	A375 melanoma cells	Sensitivity to BRAF inhibitor (PLX-4720) [51]
CRISPRi	(hCRISPRi-v2) 5	19,050	A375 melanoma cells	Resistance and sensitivity to vemurafenib [52]
CRISPRa	(hCRISPRa-v2) 5	19,050	A375 melanoma cells	Resistance and sensitivity to vemurafenib [52]
CRISPRko	(GeCKOv2) 6	19,050	A375 melanoma cells	Resistance and sensitivity to vemurafenib [52]
CRISPRa	up to 12	every coding and 4000 non-coding transcripts	K562 leukemia cells	Resistance and sensitivity to imatinib [55]
CRISPRko	total 18,315	noncoding loci surrounding CUL3, NF1, NF2	A375 melanoma cells	Resistance to vemurafenib [57]
CRISPRa	10	10,504 lncRNA TSS	A375 melanoma cells	Resistance to vemurafenib [58]
CRISPRa	(Konermann et al. 2015) 3	23,430 coding isoforms	MOLM14 AML cells	Resistance to Ara-C [59]
CRISPRa	4	14,701 lncRNA genes	MOLM14 AML cells	Resistance to Ara-C [59]
CRISPRko	(Brunello) 4 and (Gattinara) 2	19,114	Meljuso and A375 melanoma cells, OVCAR8 ovarian cells	Resistance and sensitivity to A-1331852 [56]
CRISPRko	(Brunello) 4 and (Gattinara) 2	19,114	Meljuso and A375 melanoma cells and OVCAR8 ovarian cells	Resistance and sensitivity to S63845 [56]
CRISPRko	(Brunello) 4	19,114	OVCAR8 ovarian cells and A375 melanoma cells	Resistance and sensitivity to olaparib [56]
CRISPRko	(Brunello) 4	19,114	Hap1 cells	Resistance and sensitivity to talazoparib [56]
CRISPRko	(Gattinara) 2	19,114	A375 melanoma cells	Resistance and sensitivity to talazoparib [56]

### 1.5 CRISPR-based chemogenetics for studying the noncoding genome

CRISPR-mediated chemogenics has also shown as successful in studying the noncoding genome which can affect gene regulation and disease. Sanjana et al. developed a CRISPR screen using ∼18,000 single guide RNAs targeting >700 kilobases surrounding the genes *NF1*, *NF2*, and *CUL3*, which were previously reported as involved in *BRAF* inhibitor resistance in melanoma. They found that noncoding locations that modulate drug resistance also harbor predictive hallmarks of noncoding function [57]. Another group developed a genome-scale CRISPR activation screen that targets more than 10,000 long noncoding RNA (lncRNA) transcriptional start sites to identify noncoding loci that influence a phenotype of interest. They report 11 lncRNA loci that, upon recruitment of an activator, mediate resistance to *BRAF* inhibitor. Most candidate loci appear to regulate nearby genes [58]. Both screens were done in human melanoma A375 cells. In a more recent study a global approach to integrate computational analysis of cell line pharmacogenomic datasets with functional CRISPRa screens targeting coding and non-coding genes was developed. As the authors state, this approach aimed to uncover integrated mechanisms regulating normal cellular homeostasis and disease and was applied to identifying functional lncRNAs modulating the cytotoxic effect of Ara-C, a chemotherapy agent frequently used in the treatment of AML patients. In addition to a number of coding genes and pathways previously shown to regulate the response to Ara-C treatment, their analysis also revealed a number of lncRNAs that influence response to Ara-C, and a *cis*-regulation pattern by lncRNAs on their adjacent cognate coding genes [59].

### 1.6 Arrayed CRISPR library screening

**Fig. 3:**
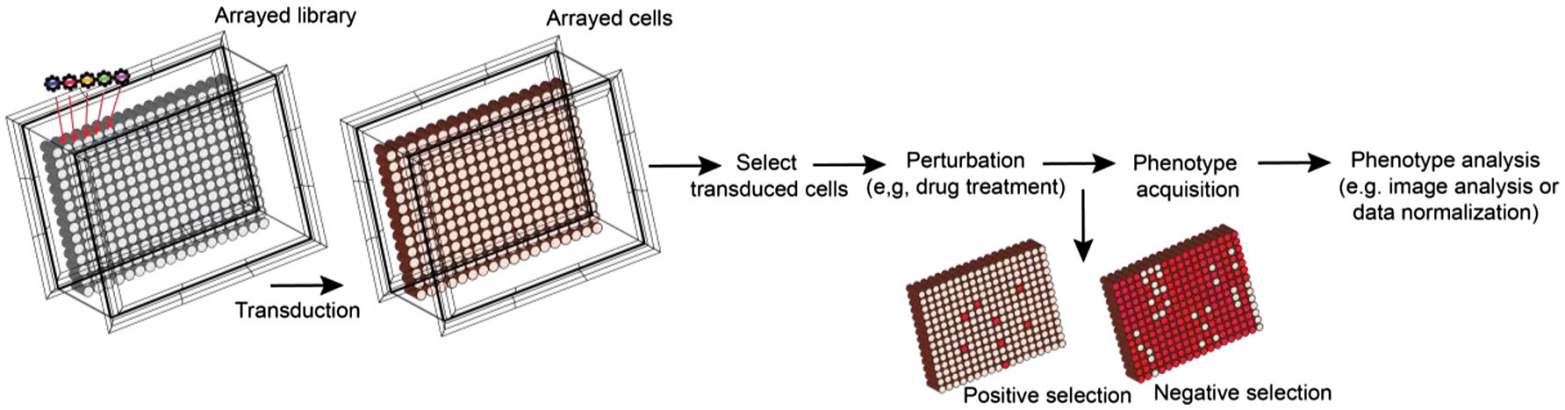
Arrayed library CRISPR screen. Arrayed libraries are generated in multi-well plates, where each well contains constructs preparation targeting an individual gene or genomic locus. Arrayed libraries are delivered to populations of cells grown in an arrayed format as well, preventing an individual cell from being transduced with multiple sgRNAs with different targets. There may be selection steps and treatments involved, but this can vary depending on the screen. Phenotypes are identified rather than necessarily being selected for (allowing for reverse genetic screening), since the sgRNA responsible for each phenotype is known based on well location in the original annotated library. The final outcome is a ranked phenotypic measure for each sgRNA delivered in the screen.

Another component of the genome editing toolbox is arrayed CRISPR library screening (Fig. 3), which is enables reverse genetic screens with a much wider utility in terms of phenotypic read-out (including fluorescence/luminescence and image-based approaches). Arrayed library screening is different from approaches using pooled libraries. Arrayed libraries are usually generated in multi-well plates, where each well contains one or more guide constructs targeting an individual gene. Such a library is delivered to cells grown in an arrayed format as well, opposite to the pooled library screen, in which a pool of cells (all grown in a single plate or set of replicate plates) is transduced with a pooled library. The arrayed set up allows exploration of complex phenotypes (e.g. subcellular localization of a fluorescent reported) rising from a number of distinct cell perturbations in parallel [60], [61], [62], [63], [64]. This screening method hasn’t yet been widely used for exploration and investigation of chemogenetic interactions, as the one-perturbation-per-well approach does not scale well, but advances in screen miniaturization could change these prospects rapidly.

### 1.7 Chemogenetics coupled with existing genomics data

Inter-institutional efforts to conduct a detailed genetic and pharmacologic characterization of a large panel of human cancer models resulted in a large-scale pharmacogenomic studies such as the Cancer Cell Line Encyclopedia (CCLE) [65], [66], [67], [68], Genomics of Drug Sensitivity in Cancer (GDSC) [69], [70], [71], and The Connectivity Map (CMap) [72], [73]. These studies provide a potential to improve cancer treatments by defining a landscape of genetic targets for therapeutic development, identifying patients who respond to these therapies, and developing a better understanding of the vulnerabilities of cancer. The CCLE, which previously was based on expression, chromosomal copy number, sequencing data and drug responses, recently expanded the characterization of cancer cell lines with RNA splicing, DNA methylation, histone H3 modification, microRNA expression and reverse-phase protein array data [65]. The CMap dataset is established on transcriptional expression data to probe relationships between diseases (cancer, neurological diseases, and infectious diseases), cell physiology, and therapeutics. With the L1000 assay for generating a large scale of expression profiles, CMap was enhanced with ∼1 million expression profiles resulting from perturbations of multiple cell types [72]. GDCS resources include ∼1000 genetically characterized human cancer cell lines screened with a wide range of therapeutic agents. Integration of drug sensitivity, RNAi, and CRISPR data with transcriptional expression profiles, copy number aberrations and mutational profiles has a potential to reveal candidate targets for cancer drugs and associated biomarkers.

### 1.8 CRISPR’s evolving role in chemogenetics

The relatively short history of chemogenetic screening in human cells reflects the ongoing competition between reagent quality, experimental scale, biological signal, and overall cost. The ultracomplex RNAi library comprises some 500,000 reagents and requires on the order of a billion cells. The most recent CRISPR chemogenetic screens use libraries of ∼70,000 unique gRNA and required fewer than 20 million cells per replicate. Importantly, vastly improved reagent quality has allowed researchers to address the question of generalizability, one of the fundamental shortcomings of in vitro screening in cell lines. The latest generation of screens was performed in multiple cell lines reflecting varied genotypes and tissues of origin, increasing confidence that the results of chemogenetic screens are not specific to the backgrounds being tested.

Lack of standardization is still an issue, however. Experimental designs include high-coverage, single-replicate experiments as well as low-coverage, multiple-replicate approaches. Bioinformatic approaches to analyzing screen data can be equally bewildering (Box 1). CRISPR reagent design continues to improve rapidly, with several groups tackling not only improved individual reagent design as well as the next step, multiplex targeting of gene pairs to identify genetic interactions [74], [75], [76], [77], [78].

Despite these issues, CRISPR-mediated chemogenomic screening clearly offers powerful insight into many aspects of drug development. Results from CRISPR-mediated loss-of-function screens for resistance against 6-thioguanine [37], etoposide [37], and vemurafenib [38] recapitulated the known mechanisms of action, validating the ability of such screens to identify targets and genetic dependencies of known drugs. Genome-scale CRISPRi and CRISPRa screens have been successful at identifying known and new pathways and complexes governing the response toxins [49]. Screens for both positive and negative regulators of drug activity have identified specific targets of uncharacterized molecules, and clarified the mechanism of action of drugs in development. With CRISPR technology rendering the human genome tractable, we have the tools to exploit genetics for drug discovery directly in human cells, as envisioned more than two decades ago.

Box 1 MAGeCK [79] identifies essential genes from genome-scale CRISPR-Cas9 knockout screens. This algorithm uses a negative binomial model to test whether sgRNA abundance differs significantly between treatment and control samples, and ranks them based on the negative binomial p-values. To rank positively or negatively selected sgRNA, gene and pathway it uses a robust ranking aggregation algorithm (RRA).

DrugZ [80] is an algorithm for identifying chemogenetic interactions from CRISPR-mediated chemogenetic screens. It calculates a fold change for each sgRNA in a treated sample relative to a control sample. It then calculates a Z-score for each guide using an empirical Bayes estimate of variance from similar sgRNAs ranked by read counts in the control cells. Guide level Z-scores are then combined into normalized gene level Z-score.

RIGER [22] is a statistical approach to enrich for on-target genes in an RNAi screen (considers the phenotypic results for the multiple shRNAs targeting the same gene). It is based on the gene set enrichment analysis (GSEA) methodology and uses similar Kolmogorov–Smirnov (KS)-based statistics to calculate gene scores from a dataset of shRNA construct profiles.

STARS [44] is a gene-ranking algorithm for genetic perturbation screens. Computing gene-level score uses the probability mass function of a binomial distribution based on the total number of perturbations targeting a gene, the within-gene rank of the perturbation, and a ratio of the rank of the within-gene perturbation over the total number of perturbations in the experiment.
